# The Impact of Short‐Term Blood Pressure Variability on All‐Cause Mortality in Patients With Acute Myocardial Infarction

**DOI:** 10.1155/crp/7788121

**Published:** 2026-07-23

**Authors:** Ying Liu, Sijia Jiang, Wen Li, Shuoyan An, Wuqiang Che, Min Wang, Jingang Zheng, Gailing Chen

**Affiliations:** ^1^ Department of Cardiology, China-Japan Friendship Hospital, Beijing, China, zryhyy.com.cn; ^2^ Graduate School, Beijing University of Chinese Medicine, Beijing, China, bucm.edu.cn; ^3^ China Academy of Chinese Medical Sciences, Institute of Chinese Materia Medica, Beijing, China, cacms.ac.cn

**Keywords:** acute myocardial infarction, blood pressure variability, MIMIC-IV, mortality

## Abstract

**Background:**

Blood pressure variability (BPV) is recognized as an independent risk factor for cardiovascular morbidity and mortality. However, findings regarding the association between the coefficient of variation (CV) of 24‐h blood pressure and mortality in patients with acute myocardial infarction (AMI) remain inconsistent. The aim of this study was to examine the association between 24‐h CV and the risk of in‐hospital, 30‐day, 1‐year, and 3‐year mortality in AMI patients.

**Methods:**

This retrospective cohort study included AMI patients who were admitted to the intensive care unit (ICU) from the MIMIC‐IV 2.0 database. The CV was calculated from blood pressure measurements taken during the first 24 h. Patients were divided into three groups according to tertiles of 24‐h blood pressure CV. The relationship between 24‐h CV and the risk of all‐cause mortality in AMI patients was analyzed using logistic regression, Cox proportional hazards regression, and Kaplan–Meier survival analyses.

**Results:**

A total of 1291 eligible AMI patients were included. Compared with Q1 (lowest CV), Q3 (highest 24‐h systolic blood pressure [SBP]‐CV) was significantly associated with an increased risk of in‐hospital (odds ratio [OR]: 3.333, 95% confidence interval [CI]: 1.725–6.439), 30‐day (hazard ratio [HR]: 1.868, 95% CI: 1.201–2.904), and 1‐year (HR: 1.420, 95% CI: 1.058–1.907) mortality. Both Q2 and Q3 of 24‐h diastolic blood pressure (DBP)–CV were significantly associated with an increased risk of in‐hospital, 30‐day, 1‐year, and 3‐year mortality. Kaplan–Meier survival curves demonstrated that patients in Q3 had significantly lower 30‐day, 1‐year, and 3‐year survival probabilities than those in other tertiles for both SBP and DBP.

**Conclusions:**

Our findings suggest that 24‐h SBP‐CV and DBP‐CV are significantly associated with both short‐ and long‐term outcomes in AMI patients. BPV may serve as a prognostic marker for all‐cause mortality in AMI patients.

## 1. Introduction

Globally, approximately 29.6% of deaths are attributed to cardiovascular diseases [1], with about one‐third resulting from acute myocardial infarction (AMI) in developed countries [2, 3]. Since the widespread use of percutaneous coronary intervention (PCI) for myocardial revascularization, the prognosis of AMI has improved [4]. However, the risk of major adverse cardiovascular events (MACE) remains high in AMI patients. Therefore, it is crucial to investigate the potential factors affecting cardiovascular events in AMI patients to further reduce mortality risk.

In recent years, the influence of blood pressure variability (BPV) on cardiovascular prognosis has garnered increasing attention. Studies have demonstrated that BPV is independently linked to the occurrence of MACE, regardless of mean blood pressure [5, 6]. BPV refers to fluctuations in blood pressure over time, influenced by hemodynamics, neurohumoral regulation, behavior, and environmental factors. Compared with mean blood pressure, BPV better reflects dynamic changes in blood pressure levels over time. BPV can be categorized into short‐term and long‐term types. Short‐term BPV includes blood pressure changes within minutes and hours, and 24‐h BPV is classified as short‐term BPV. Long‐term BPV includes blood pressure changes over several days, weeks, or months [7]. The primary indices for assessing BPV include the coefficient of variation (CV), standard deviation (SD), variation independent of the mean (VIM), and average real variability (ARV). SD and CV are among the most frequently used BPV indices in clinical research. Beyond coronary artery disease, BPV has also been increasingly recognized as a prognostic marker across the broader cardiovascular and cerebrovascular spectrum. A recent systematic review and meta‐analysis reported that increased systolic visit‐to‐visit BPV was associated with a higher incidence of stroke independent of mean blood pressure, supporting the broader clinical relevance of blood pressure fluctuation beyond average blood pressure levels [8].

The association between BPV, mortality, and cardiovascular events in AMI patients has not been extensively studied, and the results using different BPV indices are inconsistent [9–11]. A meta‐analysis [11] showed that AMI patients had an increased risk of death and cardiovascular events when their long‐term systolic blood pressure (SBP)‐SD increased. However, other studies suggest that the SD and ARV of 24‐h blood pressure are not significantly associated with cardiovascular events during hospitalization in AMI patients. Currently, there is a lack of evidence regarding how short‐term BPV affects the long‐term outcome for AMI patients.

The main objective of this study was to investigate the relationship between the first 24‐h SBP‐CV and DBP‐CV in AMI patients admitted to the ICU and the risk of short‐term and long‐term all‐cause mortality, to provide evidence for early risk stratification in AMI patients. This study focuses on short‐term BPV during the first 24 h after ICU admission in AMI patients, a period characterized by marked hemodynamic instability but limited evidence regarding prognostic BPV markers. Unlike previous studies that mainly evaluated long‐term visit‐to‐visit BPV or short‐term in‐hospital MACE, the present study assessed both short‐ and long‐term all‐cause mortality up to 3 years using a relatively large ICU‐based AMI cohort from the MIMIC‐IV database.

## 2. Materials and Methods

### 2.1. Study Design

With data obtained from the MIMIC‐IV 2.0 database, this retrospective cohort study examined AMI patients admitted to the ICU between 2008 and 2019. The database is maintained by Beth Israel Deaconess Medical Center and Massachusetts Institute of Technology. Permission for data access was obtained after completing the Collaborative Institutional Training Initiative (CITI Program) course and test (record ID: 50038981). The Beth Israel Deaconess Medical Center Institutional Review Board waived informed consent and accepted the data‐sharing effort because all patient data were anonymized.

### 2.2. Study Population

All medical information of enrolled patients was collected from the MIMIC‐IV 2.0 database.

The criteria for inclusion were outlined as follows: (1) first‐time diagnosis of AMI, which was identified by prespecified ICD‐9 and ICD‐10 diagnosis codes (ICD‐9410.xx and ICD‐10 I21.xx) and recorded as the primary diagnosis of the index hospitalization (seq_num = 1); (2) age ≥ 18 years; and (3) first‐time admission to the ICU.

The criteria for exclusion were: (1) ICU stay < 24 h, (2) < 24 eligible non‐invasive blood pressure measurements recorded during the first 24 h of ICU admission, and (3) > 20% missing covariate data.

### 2.3. Data Extraction

Data acquired from the MIMIC‐IV 2.0 database through the use of Navicat software (Navicat Premium 15, PremiumSoft, Hong Kong, Singapore) encompassed demographic information (age, sex, race); clinical conditions (including length of hospital stay, history of hypertension, diabetes); occurrence of acute heart failure, cardiogenic shock, and cardiac arrest; records of blood pressure during the initial 24 h following admission; and survival outcomes (in‐hospital, 30‐day, 1‐year, and 3‐year mortality). All disease diagnoses in the database were classified using the International Classification of Diseases, Ninth Revision, and Tenth Revision. The detailed ICD code list is provided in Supporting Table S1.

### 2.4. BPV

Noninvasive SBP and diastolic blood pressure measurements recorded during the first 24 h after ICU admission were extracted. To improve the consistency of BPV estimation, only consecutive blood pressure measurements with an interval of less than 1 h were included. Patients with fewer than 24 eligible blood pressure measurements during the first 24 h were excluded.

SD and CV are the most common measures of BPV. SD is the simplest statistical indicator used to describe variations and is significantly associated with mean blood pressure. CV reduces the influence of the absolute magnitude of mean blood pressure and reflects the relative fluctuation of blood pressure, thus enabling a comparable assessment of BPV across individuals with different baseline mean blood pressure levels. The CV was calculated according to the following formula [12]:

(1) CV=∑k=1NBPk−BP¯2/N−1BP¯.

Other BPV indices, such as SD, ARV, and VIM, may capture different aspects of blood pressure fluctuation. However, the present study focused on CV because it reflects relative variability and allows comparison across patients with different baseline blood pressure levels.

### 2.5. Outcomes

All‐cause mortality was the primary endpoint. Short‐term all‐cause mortality was defined as in‐hospital mortality and 30‐day mortality, whereas long‐term all‐cause mortality was defined as 1‐year and 3‐year mortality.

### 2.6. Statistical Analysis

Non‐normally distributed continuous variables are presented as medians and interquartile ranges. Intergroup comparisons were performed using the Kruskal–Wallis test. The Chi‐squared test was used to assess categorical variables. Cox proportional hazards and logistic regression analysis were employed to evaluate the relationship between 24‐h BPV and all‐cause mortality. Odds ratio (OR) and 95% confidence interval (CI) were reported for logistic regression analyses. The hazard ratio (HR) and 95% CI were reported for Cox proportional hazards regression analyses. Variables related to the risk of death were explored using univariable analysis (*p* < 0.1), and statistically significant variables were included in the multivariable analysis as covariates. Univariable analysis showed that age, hypertension, cardiogenic shock, acute heart failure, cardiac arrest, and PCI were associated with mortality risk in AMI patients (*p* < 0.1). Considering that sex and diabetes mellitus are also important clinical covariates, age, hypertension, acute heart failure, cardiogenic shock, cardiac arrest, PCI, sex, and diabetes mellitus were included in the multivariable regression model. All the covariates and independent variables were subjected to collinearity tests. The cumulative risk of death for the three groups of 24‐h SBP‐CV and 24‐h DBP‐CV was represented by the Kaplan–Meier curve. Statistical analysis was conducted using SPSS (IBM SPSS, Inc., Armonk, NY, USA). Graphs were created using R statistical software. The statistical significance threshold was set at a two‐sided *p*‐value of less than 0.05.

## 3. Results

### 3.1. Baseline Characteristics

**FIGURE 1:**
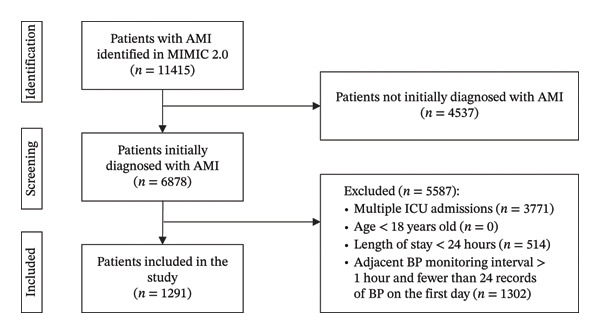
Flowchart for screening patients. AMI, acute myocardial infarction; BP, blood pressure; ICU, intensive care unit.

A total of 11,415 AMI patients were extracted from the MIMIC‐IV 2.0 database. Based on the criteria for inclusion and exclusion, 1291 patients were included in this study (Figure 1). The median age of the participants in the study was 71 years; 63.2% of the patients were male, 43.5% had a history of hypertension, and 16.3% had a history of diabetes. A proportion of 47.6% of the patients underwent PCI. Acute heart failure, cardiac arrest, and cardiogenic shock occurred in 17.7%, 4.6%, and 11.7% of the patients during hospitalization, respectively.

In this study, three groups of patients were identified using the tertiles of CV, an index of BPV in the first 24 h following admission to the ICU. The baseline characteristics of the groups are presented in Table 1. When grouped according to SBP‐CV tertiles, no significant differences in comorbidities were seen among the groups. However, age, sex, and random blood glucose levels varied considerably among groups (*p* < 0.05) (Table 1). When grouped according to DBP‐CV tertiles, there were statistically notable differences in sex, age, hemoglobin, platelets, and troponin T (*p* < 0.05) (Table 1). Patients in the highest SBP‐CV and DBP‐CV tertiles had the highest median age. In the highest SBP‐CV tertile, male patients remained more common than female patients, although the proportion of female patients increased across SBP‐CV tertiles. In contrast, female patients accounted for the majority in the highest DBP‐CV tertile.

**TABLE 1:** Baseline characteristics of the patients.

	All patients	Tertiles of SBP‐CV				Tertiles of DBP‐CV			
		Q1 (≤ 0.0925)	Q2 (0.0925–0.1234)	Q3 (≥ 0.1234)	*p*	Q1 (≤ 0.1255)	Q2 (0.1255–0.1749)	Q3 (≥ 0.1749)	*p*
*N*	1291	429	432	430		431	430	430	
Age, years, median (IQR)	71.0 (61.0, 81.0)	70.0 (60.0, 78.0)	70.0 (61.0, 81.0)	75.0 (65.0, 84.0)	< 0.001	67.0 (57.0, 75.0)	70.0 (60.0, 79.0)	78.0 (69.0, 85.0)	< 0.001
Female sex, *n* (%)	475 (36.8)	122 (28.44)	160 (37.04)	193 (44.88)	< 0.001	96 (22.27)	150 (34.88)	229 (53.26)	< 0.001
Race, *n* (%)					0.986				0.910
Asian	24 (1.9)	8 (1.86)	7 (1.62)	9 (2.09)		10 (2.32)	5 (1.16)	9 (2.09)	
White	833 (64.5)	273 (63.64)	284 (65.74)	276 (64.19)		278 (64.50)	280 (65.12)	275 (63.95)	
Black	90 (7.0)	33 (7.69)	28 (6.48)	29 (6.74)		31 (7.19)	28 (6.51)	31 (7.21)	
Other	344 (26.6)	115 (26.81)	113 (26.16)	116 (26.98)		112 (25.99)	117 (27.21)	115 (26.74)	
Comorbidities, *n* (%)									
Hypertension	562 (43.5)	168 (39.16)	204 (47.22)	190 (44.19)	0.055	182 (42.23)	188 (43.72)	192 (44.65)	0.770
Diabetes	210 (16.3)	78 (18.18)	56 (12.96)	76 (17.67)	0.073	74 (17.17)	64 (14.88)	72 (16.74)	0.627
Acute heart failure	228 (17.7)	79 (18.41)	71 (16.44)	78 (18.14)	0.711	78 (18.10)	78 (18.14)	72 (16.74)	0.830
Cardiogenic shock	151 (11.7)	41 (9.56)	47 (10.88)	63 (14.65)	0.055	46 (10.67)	55 (12.79)	50 (11.63)	0.626
Cardiac arrest	59 (4.6)	15 (3.50)	21 (4.86)	23 (5.35)	0.403	22 (5.10)	20 (4.65)	17 (3.95)	0.718
Laboratory parameters									
HGB (g/dL)	12.1 (10.3, 13.5)	12.4 (10.3, 13.7)	12.0 (10.3, 13.4)	11.9 (10.1, 13.4)	0.254	12.4 (10.8, 13.8)	12.3 (10.4, 13.8)	11.4 (9.7, 12.9)	< 0.001
Platelet (^∗^10^9^/L)	215.0 (174.0, 267.0)	214.0 (173.0, 269.0)	215.5 (175.0, 261.0)	218.0 (175.5, 275.5)	0.418	208.0 (172.0, 262.0)	214.5 (171.0, 265.8)	224.0 (178.0, 277.0)	0.030
TNT (ng/mL)	0.97 (0.31, 3.29)	1.08 (0.34, 3.20)	1.10 (0.36, 3.43)	0.83 (0.25, 3.21)	0.127	1.27 (0.43, 4.06)	0.97 (0.34, 3.62)	0.77 (0.24, 2.43)	< 0.001
Cr (mg/dL)	1.1 (0.8, 1.5)	1.0 (0.8, 1.4)	1.1 (0.8, 1.5)	1.1 (0.8, 1.6)	0.900	1.0 (0.8, 1.5)	1.0 (0.8, 1.5)	1.1 (0.8, 1.6)	0.239
GLU (mg/dL)	133.0 (109.0, 179.0)	129.0 (107.0, 171.0)	131.0 (109.0, 175.0)	140.0 (116.0, 195.5)	0.003	131.0 (107.0, 178.0)	133.0 (109.3, 179.0)	137.0 (112.0, 185.5)	0.113
PCI, *n* (%)	614 (47.6)	199 (46.39)	205 (47.45)	210 (48.84)	0.771	192 (44.55)	216 (50.23)	206 (47.91)	0.244

*Note:* TNT: troponin T; Cr: creatinine; GLU: glucose; HGB: hemoglobin.

Abbreviations: CV, coefficient of variation; DBP, diastolic blood pressure; PCI, percutaneous coronary intervention; SBP, systolic blood pressure.

### 3.2. Relationship Between 24‐h BPV and Mortality in AMI Patients

In this study, the in‐hospital mortality rate of AMI patients was 6.5%, the 30‐day mortality rate was 10.2%, the 1‐year mortality rate was 23.0%, and the 3‐year mortality rate was 27.6%. Comparing the mortality rates among the groups, we found that short‐ and long‐term mortality rates increased with increasing SBP‐CV and DBP‐CV (Table 2). No evidence of nonlinearity was observed for the associations of SBP‐CV and DBP‐CV with short‐ and long‐term mortality (*p* for nonlinearity > 0.05).

**TABLE 2:** Comparison of all‐cause mortality based on the CV group.

Outcomes	All patients	Tertiles of SBP‐CV				Tertiles of DBP‐CV			
		Q1 (≤ 0.0925)	Q2 (0.0925–0.1234)	Q3 (≥ 0.1234)	*p*	Q1 (≤ 0.1255)	Q2 (0.1255–0.1749)	Q3 (≥ 0.1749)	*p*
*N*	1291	429	432	430		431	430	430	
In‐hospital mortality, *n* (%)	84 (6.5)	16 (3.73)	20 (4.63)	48 (11.16)	< 0.001	16 (3.71)	32 (7.44)	36 (8.37)	0.013
30‐day mortality, *n* (%)	132 (10.2)	31 (7.23)	33 (7.64)	68 (15.81)	< 0.001	24 (5.57)	47 (10.93)	61 (14.19)	< 0.001
1‐year mortality, *n* (%)	297 (23.0)	75 (17.48)	92 (21.30)	130 (30.23)	< 0.001	56 (12.99)	105 (24.42)	136 (31.63)	< 0.001
3‐year mortality, *n* (%)	356 (27.6)	93 (21.68)	116 (26.85)	147 (34.19)	< 0.001	70 (16.24)	122 (28.37)	164 (38.14)	< 0.001

Abbreviations: CV, coefficient of variation; DBP, diastolic blood pressure; SBP, systolic blood pressure.

In‐hospital mortality was analyzed using a multivariable logistic regression model, and the 30‐day, 1‐year, and 3‐year mortality rates were analyzed using the Cox proportional hazards model.

### 3.3. Relationship Between SBP‐CV and Mortality

The results of the multivariable analysis showed that the Q3 group with the highest SBP‐CV was associated with increased in‐hospital (OR: 3.333, 95% CI: 1.725–6.439), 30‐day (HR: 1.868, 95% CI: 1.201–2.904), and 1‐year (HR: 1.420, 95% CI: 1.058–1.907) mortality rates compared with the Q1 group with the lowest CV (*p* < 0.05) (Table 3).

**TABLE 3:** Multivariable analysis of the association between SBP‐CV and mortality.

Outcomes	Groups	Mortality, *n* (%)	Univariable model		Multivariable model	
			OR/HR (95% CI)	*p*	OR/HR (95% CI)	*p*
In‐hospital mortality	Q1	16 (3.73)	Reference	—	Reference	—
	Q2	20 (4.63)	1.253 (0.640–2.452)	0.510	1.258 (0.597–2.654)	0.546
	Q3	48 (11.16)	3.243 (1.811–5.808)	< 0.001	3.333 (1.725–6.439)	< 0.001

30‐day mortality	Q1	31 (7.23)	Reference	—	Reference	—
	Q2	33 (7.64)	1.061 (0.650–1.733)	0.812	1.045 (0.636–1.716)	0.861
	Q3	68 (15.81)	2.300 (1.504–3.518)	< 0.001	1.868 (1.201–2.904)	0.006

1‐year mortality	Q1	75 (17.48)	Reference	—	Reference	—
	Q2	92 (21.30)	1.236 (0.911–1.677)	0.173	1.186 (0.872–1.615)	0.277
	Q3	130 (30.23)	1.900 (1.430–2.525)	< 0.001	1.420 (1.058–1.907)	0.020

3‐year mortality	Q1	93 (21.68)	Reference	—	Reference	—
	Q2	116 (26.85)	1.267 (0.964–1.664)	0.089	1.218 (0.924–1.605)	0.161
	Q3	147 (34.19)	1.756 (1.354–2.277)	< 0.001	1.300 (0.993–1.701)	0.056

Abbreviations: 95% CI, 95% confidence interval; HR, hazard ratio; OR, odds ratio.

### 3.4. Relationship Between DBP‐CV and Mortality

Both Q2 and Q3 of DBP‐CV were significantly associated with increased in‐hospital ([OR: 2.608, 95% CI: 1.289–5.276] [OR: 2.671, 95% CI: 1.307–5.459]), 30‐day ([HR: 2.052, 95% CI: 1.241–3.393] [HR: 2.352, 95% CI: 1.410–3.923]), 1‐year ([HR: 1.849, 95% CI: 1.329–2.571] [HR: 1.850, 95% CI: 1.324–2.583]), and 3‐year ([HR: 1.751, 95% CI: 1.299–2.360] [HR: 1.817, 95% CI: 1.346–2.455]) mortality rates (*p* < 0.05) (Table 4).

**TABLE 4:** Multivariable analysis of the association between DBP‐CV and mortality.

Outcomes	Groups	Mortality, *n* (%)	Univariable model		Multivariable model	
			OR/HR (95% CI)	*p*	OR/HR (95% CI)	*p*
In‐hospital mortality	Q1	16 (3.71)	Reference	—	Reference	—
	Q2	32 (7.44)	2.085 (1.127–3.860)	0.019	2.608 (1.289–5.276)	0.008
	Q3	36 (8.37)	2.370 (1.294–4.339)	0.005	2.671 (1.307–5.459)	0.007

30‐day mortality	Q1	24 (5.57)	Reference	—	Reference	—
	Q2	47 (10.93)	2.007 (1.228–3.282)	0.005	2.052 (1.241–3.393)	0.005
	Q3	61 (14.19)	2.657 (1.657–4.261)	< 0.001	2.352 (1.410–3.923)	0.001

1‐year mortality	Q1	56 (12.99)	Reference	—	Reference	—
	Q2	105 (24.42)	1.988 (1.438–2.750)	< 0.001	1.849 (1.329–2.571)	< 0.001
	Q3	136 (31.63)	2.684 (1.966–3.664)	< 0.001	1.850 (1.324–2.583)	< 0.001

3‐year mortality	Q1	70 (16.24)	Reference	—	Reference	—
	Q2	122 (28.37)	1.879 (1.400–2.520)	< 0.001	1.751 (1.299–2.360)	< 0.001
	Q3	164 (38.14)	2.665 (2.014–3.526)	< 0.001	1.817 (1.346–2.455)	< 0.001

Abbreviations: 95% CI, 95% confidence interval; HR, hazard ratio; OR, odds ratio.

**FIGURE 2:**
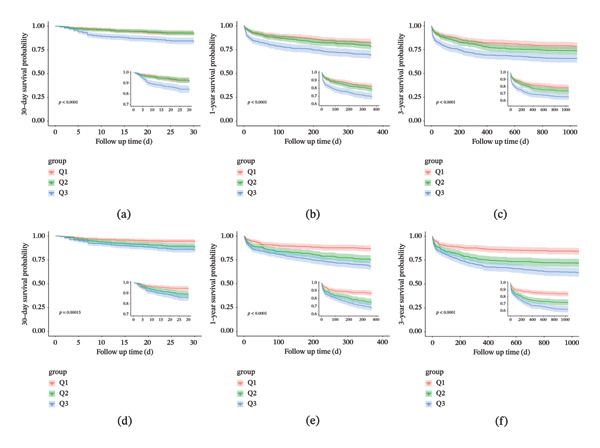
Kaplan–Meier survival curves of patients according to the systolic (SBP) and diastolic blood pressure (DBP) coefficient of variation (CV).

The Kaplan–Meier survival curves showed that, regardless of SBP or DBP, patients in the Q3 group with the highest CV for both SBP and DBP had significantly lower probabilities of survival at 30 days, 1 year, and 3 years than those in the other tertile groups (Figure 2).

## 4. Discussion

With the widespread adoption of revascularization therapy, AMI patients now have an improved prognosis; however, the risk of MACE remains high [13]. Therefore, identifying factors that affect the prognosis of AMI patients has become a critical focus. In this study, we investigated the association between short‐term BPV and all‐cause mortality in AMI patients. Higher 24‐h SBP‐CV was associated with increased risks of in‐hospital, 30‐day, and 1‐year mortality in AMI patients. Additionally, increased 24‐h DBP‐CV was associated with a higher risk of both short‐term and long‐term mortality. Given the retrospective observational design, causality cannot be inferred. Elevated BPV may represent a marker of underlying hemodynamic instability, autonomic dysfunction, impaired vascular regulation, or greater disease severity rather than a direct causal determinant of mortality.

Recent studies have confirmed that hypertension‐related cardiovascular risk is determined not only by mean blood pressure levels, but also by BPV. Regardless of the time frame, increased BPV has been related to the onset, progression, and severity of damage to target organs such as the heart, blood vessels, brain, and kidneys. It is also linked with a higher risk of cardiovascular events and all‐cause mortality, independent of elevated mean blood pressure levels [11, 14]. The prognostic significance of BPV is not limited to AMI. Recent evidence suggests that BPV contributes to risk stratification across the cardiovascular and cerebrovascular spectrum, including stroke, coronary artery disease, heart failure, renal outcomes, and mortality [8]. In addition, short‐term BPV has been associated with choroidal–retinal structural alterations in hypertensive patients [15], and BPV has been increasingly recognized in relation to subclinical renal damage and adverse renal outcomes [16]. These findings support the concept that BPV may reflect systemic vascular dysfunction, impaired cardiovascular regulation, microvascular damage, and target‐organ vulnerability rather than isolated blood pressure fluctuation. Early studies on BPV and cardiovascular events primarily focused on hypertensive populations. In recent years, research has progressively demonstrated the associations of BPV with cardiovascular events, target‐organ damage, and mortality risk in nonhypertensive populations, such as those with diabetes mellitus, obstructive sleep apnea, and chronic kidney disease (CKD) [17, 18]. Recently, researchers have also begun to focus on how BPV affects AMI patients’ prognoses.

However, the impact of BPV on cardiovascular events remains controversial. Independent of mean BP, BPV predicts cardiovascular events, according to most research. Compared to mean BP, long‐term BPV may be a better predictor of cardiovascular events. Furthermore, long‐term BPV is significantly associated with the risk of all‐cause mortality in patients with coronary artery disease and stroke [19, 20]. Other research, however, has demonstrated that there is no discernible link between long‐term BPV and the risk of cardiovascular events [21]. In contrast, short‐term BPV is more predictive of mortality in elderly hypertensive patients [22], and 24‐h BPV is significantly associated with a higher risk of early mortality after coronary artery bypass grafting [23]. In addition to its association with cardiovascular events, BPV is also associated with renal events. Jhee [24] found that 24‐h SBP‐ARV was associated with an elevated likelihood of all‐cause death, cardiovascular disease, and decreased renal function in CKD patients. Mallamaci [25] concluded that only long‐term systolic BPV is related to the risk of cardiovascular events and death in patients with CKD.

Currently, there are few studies on the relationship between BPV and AMI prognosis, and the results are mixed. A prospective study [26] suggested that the 24‐h BPV index SDdn was significantly associated with the risk of all‐cause mortality during hospitalization in patients with acute coronary syndrome (ACS) and was the only independent predictor of MACE during hospitalization in ACS patients. However, another study [9] showed that 24‐h BPV‐SD and BPV‐ARV in the first 24 h of admission were not significantly associated with MACE during hospitalization in AMI patients. Compared with short‐term BPV, Choo [10] found that long‐term BPV was linked to the long‐term prognosis of AMI patients after PCI, and DBP VIM was an independent predictor of all‐cause mortality and MACE. In this study, we investigated the relationship between 24‐h BPV‐CV and the risk of all‐cause mortality in a larger sample and found that elevated 24‐h SBP‐CV was significantly associated with a greater likelihood of all‐cause mortality during hospitalization in AMI patients. It was also significantly associated with increased all‐cause mortality at 30 days and 1 year. Moreover, there was a significant association found between raised 24‐h DBP‐CV and higher all‐cause mortality over the short and long terms. Although the BPV indices used in this study differ from those in previous studies, similar trends were observed [10], suggesting that short‐term BPV is strongly associated with MACE during hospitalization in AMI patients.

Owing to the different BPV indices used, results regarding BPV and cardiovascular outcomes are not entirely consistent across studies. Although a few studies have shown an association among SD, CV, ARV, and VIM [10], the exact relationship among the different BPV indicators has yet to be clarified. Several studies using different BPV indices have shown similar trends in BPV and cardiovascular outcomes. Previous studies have found a relationship between 24‐h and long‐term CV [27]. The relationship between different BPV indices deserves more consideration.

Animal experiments and clinical trials have shown that changes in BPV are related to arterial remodeling, arteriosclerosis, vascular injury, and endothelial dysfunction [28–31]. Additionally, BPV, arterial remodeling, and sclerosis interact with each other: greater BPV can promote arterial remodeling and sclerosis, while arterial remodeling and sclerosis can also increase BPV [32]. Endothelial dysfunction is present in most AMI patients, leading to increased BPV, which is an important factor in impaired cardiac structure and function in AMI patients [33, 34], and may be a potential reason for the effect of BPV on prognosis in AMI patients.

This research has several limitations. First, although AMI was restricted to the primary diagnosis of the index hospitalization, some covariates were derived from hospitalization‐level ICD‐coded diagnosis records. Therefore, certain acute conditions, such as acute heart failure, cardiogenic shock, and cardiac arrest, may have represented complications occurring during hospitalization rather than conditions definitively present before ICU admission. Second, residual confounding cannot be excluded because several clinically important variables were not included in the present analysis. Although MIMIC‐IV contains medication prescription and administration data as well as ICU infusion records, beta‐blocker use and vasopressor exposure were not extracted because medication exposure during the first 24 h after ICU admission requires time‐dependent definitions and may be strongly influenced by hemodynamic instability and treatment indications. PCI status was included in the multivariable model; however, the exact timing of revascularization could not be reliably determined from the structured procedure or billing data in MIMIC‐IV v2.0. In addition, direct measures of infarct severity, such as infarct size, Killip class, left ventricular ejection fraction, culprit vessel, and TIMI flow, were not available as standardized structured variables. Therefore, residual confounding by treatment exposure, disease severity, and revascularization timing may remain. Third, as a retrospective study, there may be selection bias. Fourth, the database may contain blood pressure measurements spaced at varying intervals. Although only noninvasive blood pressure measurements were included and consecutive blood pressure measurements were restricted to intervals of less than 1 h, the measurement frequency was not fully standardized because of the retrospective nature of the database. Therefore, potential measurement bias cannot be completely excluded. Finally, we only explored the effect of the first 24‐h BPV on prognosis after ICU admission and did not examine the possible impact of long‐term BPV on prognosis.

In conclusion, we found that AMI patients with higher SBP‐CV within the first 24 h after ICU admission had a higher risk of in‐hospital, 30‐day, and 1‐year mortality, and that patients with higher 24‐h DBP‐CV had a significantly higher risk of both short‐term and long‐term all‐cause mortality. Compared with previous studies, our findings further suggest that 24‐h BP‐CV may be associated with long‐term prognosis in AMI patients. Based on the results of this investigation, BPV may serve as a potential prognostic marker for all‐cause mortality in AMI patients. Future prospective studies are needed to clarify the causal relevance of BPV and to determine whether BPV is a modifiable therapeutic target in AMI patients.

## Funding

No funding was received for this study.

## Ethics Statement

The requirement of ethical approval for this study was waived by the Institutional Review Board of China‐Japan Friendship Hospital because the data were accessed from MIMIC (a publicly available database). The need for written informed consent was waived by the Institutional Review Board of China‐Japan Friendship Hospital due to the retrospective nature of the study. All methods were performed in accordance with the relevant guidelines and regulations.

## Conflicts of Interest

The authors declare no conflicts of interest.

## Supporting Information

Additional supporting information can be found online in the Supporting Information section.

## Supporting information


**Supporting Information** Supporting Table S1. ICD code definitions used for AMI and covariates, Supporting Table S2. Candidate covariates considered for model building. These materials provide further details supporting the main findings of the study.

## Data Availability

The data that support the findings of this study are openly available in MIMIC at https://physionet.org/content/mimiciv/2.0/.

## References

[1] Lozano R. , Naghavi M. , Foreman K. et al., Global and Regional Mortality From 235 Causes of Death for 20 Age Groups in 1990 and 2010: A Systematic Analysis for the Global Burden of Disease Study 2010, Lancet. (December 15 2012) 380, no. 9859, 2095–2128, 10.1016/s0140-6736(12)61728-0. 23245604 PMC10790329

[2] Yeh R. W. , Sidney S. , Chandra M. , Sorel M. , Selby J. V. , and Go A. S. , Population Trends in the Incidence and Outcomes of Acute Myocardial Infarction, New England Journal of Medicine. (2010) 362, no. 23, 2155–2165, 10.1056/nejmoa0908610. 20558366

[3] Reed G. W. , Rossi J. E. , and Cannon C. P. , Acute Myocardial Infarction, Lancet. (January 14 2017) 389, no. 10065, 197–210, 10.1016/s0140-6736(16)30677-8. 27502078

[4] Bhatt D. L. , Lopes R. D. , and Harrington R. A. , Diagnosis and Treatment of Acute Coronary Syndromes: A Review, JAMA. (2022) 327, no. 7, 662–675, 10.1001/jama.2022.0358. 35166796

[5] Bilo G. , Dolan E. , O’Brien E. et al., The Impact of Systolic and Diastolic Blood Pressure Variability on Mortality is Age Dependent: Data From the Dublin Outcome Study, European Journal of Preventive Cardiology. (2019) . 10.1177/2047487319872572 31510817

[6] Palatini P. , Saladini F. , Mos L. et al., Short-Term Blood Pressure Variability Outweighs Average 24-h Blood Pressure in the Prediction of Cardiovascular Events in Hypertension of the Young, Journal of Hypertension. (2019) 37, no. 7, 1419–1426, 10.1097/hjh.0000000000002074. 30882599

[7] Parati G. , Ochoa J. E. , Lombardi C. , and Bilo G. , Blood Pressure Variability: Assessment, Predictive Value, and Potential as a Therapeutic Target, Current Hypertension Reports. (April 2015) 17, no. 4, 10.1007/s11906-015-0537-1. 25790801

[8] Stamou E. , Iliakis P. , Konstantinidis D. et al., Association of Blood Pressure Variability and Incidence of Stroke: A Systematic Review and Meta-Analysis, Journal of Hypertension. (2025) 43, no. 11, 1764–1772, 10.1097/hjh.0000000000004129. 40986655

[9] Harefa W. I. P. , Muhadi , Muhadi et al., The Association Between 24-h Blood Pressure Variability and Major Adverse Cardiac Events (MACE) in Hospitalized Patients With Acute Myocardial Infarction: A Retrospective Cohort Study, Egypt Heart J. (October 14 2021) 73, no. 1, 10.1186/s43044-021-00213-1. PMC8517047 34648099

[10] Choo E. H. , Mok J. S. , Chung W. B. et al., Visit-to-Visit Blood Pressure Variability and Mortality and Cardiovascular Outcomes After Acute Myocardial Infarction, Journal of Human Hypertension. (November 2022) 36, no. 11, 960–967, 10.1038/s41371-021-00594-5. 34518618

[11] Stevens S. L. , Wood S. , Koshiaris C. et al., Blood Pressure Variability and Cardiovascular Disease: Systematic Review and Meta-Analysis, BMJ. (August 9 2016) 354, 10.1136/bmj.i4098. PMC4979357 27511067

[12] Park S. , Lee H. C. , Jung C. W. et al., Intraoperative Arterial Pressure Variability and Postoperative Acute Kidney Injury, Clinical Journal of the American Society of Nephrology. (January 7 2020) 15, no. 1, 35–46, 10.2215/cjn.06620619. 31888922 PMC6946069

[13] Upur H. , Li J. L. , Zou X. G. et al., Short and Long-Term Prognosis of Admission Hyperglycemia in Patients With and Without Diabetes After Acute Myocardial Infarction: A Retrospective Cohort Study, Cardiovascular Diabetology. (June 23 2022) 21, no. 1, 10.1186/s12933-022-01550-4. PMC9229884 35739511

[14] Parati G. , Ochoa J. E. , Lombardi C. , and Bilo G. , Assessment and Management of Blood-Pressure Variability, Nature Reviews Cardiology. (2013) 10, no. 3, 143–155, 10.1038/nrcardio.2013.1. 23399972

[15] Carollo C. , Vadalà M. , Ferrara M. et al., Relationship Between Short-Term Blood Pressure Variability and Choroidal-Retinal Thicknesses Assessed by Optical Coherence Tomography in Hypertensive Subjects, Journal of Personalized Medicine. (2024) 14, no. 12, 10.3390/jpm14121123. PMC11677911 39728036

[16] Carollo C. , Sorce A. , Vario M. G. et al., Relationship Between Subclinical Renal Damage and Maximum Rate of Blood Pressure Variation Assessed by Fourier Analysis of 24-h Blood Pressure Curve in Patients With Essential Hypertension, Life (Basel). (2025) 15, no. 7, 10.3390/life15071149. PMC12299583 40724654

[17] Pengo M. F. , Ratneswaran C. , Berry M. et al., Effect of Continuous Positive Airway Pressure on Blood Pressure Variability in Patients With Obstructive Sleep Apnea, Journal of Clinical Hypertension. (2016) 18, no. 11, 1180–1184, 10.1111/jch.12845. 27251875 PMC8031566

[18] Fletcher R. A. , Arnott C. , Rockenschaub P. et al., Canagliflozin, Blood Pressure Variability, and Risk of Cardiovascular, Kidney, and Mortality Outcomes: Pooled Individual Participant Data From the CANVAS and CREDENCE Trials, Journal of American Heart Association. (July 4 2023) 12, no. 13, 10.1161/jaha.122.028516. PMC10356084 37345834

[19] Dasa O. , Smith S. M. , Howard G. et al., Association of 1-Year Blood Pressure Variability With Long-Term Mortality Among Adults With Coronary Artery Disease: A Post Hoc Analysis of a Randomized Clinical Trial, JAMA Network Open. (2021) 4, no. 4, 10.1001/jamanetworkopen.2021.8418. PMC8085725 33914047

[20] Goldbourt U. and Grossman E. , Blood Pressure Variability at Midlife is Associated With all-Cause, Coronary Heart Disease and Stroke Long Term Mortality, Journal of Hypertension. (2020) 38, no. 9, 1722–1728, 10.1097/hjh.0000000000002447. 32371770

[21] Mancia G. , Facchetti R. , Parati G. , and Zanchetti A. , Visit-to-Visit Blood Pressure Variability, Carotid Atherosclerosis, and Cardiovascular Events in the European Lacidipine Study on Atherosclerosis, Circulation. (2012) 126, no. 5, 569–578, 10.1161/circulationaha.112.107565. 22761453

[22] Chowdhury E. K. , Wing L. M. H. , Jennings G. L. R. , Beilin L. J. , and Reid C. M. , Visit-to-Visit (Long-Term) and Ambulatory (Short-Term) Blood Pressure Variability to Predict Mortality in an Elderly Hypertensive Population, Journal of Hypertension. (2018) 36, no. 5, 1059–1067, 10.1097/hjh.0000000000001652. 29266060

[23] Zhou Z. , Chen J. , Fu G. et al., Association of Post-Operative Systolic Blood Pressure Variability With Mortality After Coronary Artery Bypass Grafting, Frontiers in Cardiovascular Medicine. (2021) 8, 10.3389/fcvm.2021.717073. PMC8387866 34458342

[24] Jhee J. H. , Seo J. , Lee C. J. et al., Ambulatory Blood Pressure Variability and Risk of Cardiovascular Events, all-Cause Mortality, and Progression of Kidney Disease, Journal of Hypertension. (2020) 38, no. 9, 1712–1721, 10.1097/hjh.0000000000002477. 32516289

[25] Mallamaci F. , Tripepi G. , D’Arrigo G. et al., Blood Pressure Variability, Mortality, and Cardiovascular Outcomes in CKD Patients, Clinical Journal of the American Society of Nephrology. (2019) 14, no. 2, 233–240, 10.2215/CJN.04030318. 30602461 PMC6390905

[26] Hassan A. K. M. , Abd-El Rahman H. , Mohsen K. , and Dimitry S. R. , Impact of In-Hospital Blood Pressure Variability on Cardiovascular Outcomes in Patients With Acute Coronary Syndrome, Journal of Clinical Hypertension. (2017) 19, no. 12, 1252–1259, 10.1111/jch.13107. 29105946 PMC8030841

[27] Mancia G. , Facchetti R. , Quarti-Trevano F. , Dell’Oro R. , Cuspidi C. , and Grassi G. , Comparison Between Visit-to-Visit Office and 24-h Blood Pressure Variability in Treated Hypertensive Patients, Journal of Hypertension. (2024) 42, no. 1, 161–168, 10.1097/hjh.0000000000003582. 37850964 PMC10712992

[28] Messerli F. H. , Rimoldi S. F. , and Bangalore S. , Blood Pressure Variability and Arterial Stiffness-Chicken or Egg?, JAMA Cardiology. (2019) 4, no. 10, 10.1001/jamacardio.2019.2730. 31389996

[29] Clark D. I. I. I. , Nicholls S. J. , St John J. et al., Visit-to-Visit Blood Pressure Variability, Coronary Atheroma Progression, and Clinical Outcomes, JAMA Cardiology. (2019) 4, no. 5, 437–443, 10.1001/jamacardio.2019.0751. 30969323 PMC6537804

[30] Zhou T. L. , Henry R. M. A. , Stehouwer C. D. A. , van Sloten T. T. , Reesink K. D. , and Kroon A. A. , Blood Pressure Variability, Arterial Stiffness, and Arterial Remodeling, Hypertension. (2018) 72, no. 4, 1002–1010, 10.1161/hypertensionaha.118.11325. 30354707

[31] Boardman H. , Lewandowski A. J. , Lazdam M. et al., Aortic Stiffness and Blood Pressure Variability in Young People: A Multimodality Investigation of Central and Peripheral Vasculature, Journal of Hypertension. (2017) 35, no. 3, 513–522, 10.1097/hjh.0000000000001192. 27846043 PMC5278891

[32] Miyauchi S. , Nagai M. , Dote K. et al., Visit-to-Visit Blood Pressure Variability and Arterial Stiffness: Which Came First: the Chicken or the Egg?, Current Pharmaceutical Design. (2019) 25, no. 6, 685–692, 10.2174/1381612825666190329122024. 30931845

[33] Parati G. , Stergiou G. S. , Dolan E. , and Bilo G. , Blood Pressure Variability: Clinical Relevance and Application, Journal of Clinical Hypertension. (2018) 20, 1133–1137, 10.1111/jch.13304. 30003704 PMC8030809

[34] Kishi T. , Baroreflex Failure and Beat-to-Beat Blood Pressure Variation, Hypertension Research. (2018) 41, no. 8, 547–552, 10.1038/s41440-018-0056-y. 29880837

