# Supplementary material for: The Impact of Short‐Term Blood Pressure Variability on All‐Cause Mortality in Patients With Acute Myocardial Infarction
Source: Cardiol Res Pract. 2026 Jul 23;2026:7788121. doi: 10.1155/crp/7788121 (PMC13396695; doi:10.1155/crp/7788121)
Supplement: Supplementary file 1 — Supporting Information Supporting Table S1. ICD code definitions used for AMI and covariates, Supporting Table S2. Candidate covariates considered for model building. These materials provide further details supporting the main findings of the study. [file CRP-2026-7788121-s001.docx]

Supplementary Table S1. ICD code definitions used for AMI and covariates

| Variable | Definition source | ICD-9 codes | ICD-10 codes |
| --- | --- | --- | --- |
| Acute myocardial infarction (AMI) | diagnoses_icd, restricted to primary diagnosis (seq_num = 1) | 41000, 41001, 41002, 41010, 41011, 41012, 41020, 41021, 41022, 41030, 41031, 41032, 41040, 41041, 41042, 41050, 41051, 41052, 41060, 41061, 41062, 41070, 41071, 41072, 41080, 41081, 41082, 41090, 41091, 41092 | I21, I210, I2101, I2102, I2109, I211, I2111, I2119, I212, I2121, I2129, I213, I214, I219 |
| Hypertension | diagnoses_icd during index hospitalization | 4010, 4011, 4019, 40501, 40509, 40511, 40519, 40591, 40599 | I10, I15, I150, I151, I152, I158, I159 |
| Diabetes mellitus | diagnoses_icd during index hospitalization | None specified in the SQL logic provided | E10, E1010, E1011, E102, E1021, E1022, E1029, E103, E1031, E10311, E10319, E1032, E10321, E103211, E103212, E103213, E103219, E10329, E103291, E103292, E103293, E103299, E1033, E10331, E103311, E103312, E103313, E103319, E10339, E103391, E103392, E103393, E103399, E1034, E10341, E103411, E103412, E103413, E103419, E10349, E103491, E103492, E103493, E103499, E1035, E10351, E103511, E103512, E103513, E103519, E10352, E103521, E103522, E103523, E103529, E10353, E103531, E103532, E103533, E103539, E10354, E103541, E103542, E103543, E103549, E10355, E103551, E103552, E103553, E103559, E10359, E103591, E103592, E103593, E103599, E1036, E1037, E1037X1, E1037X2, E1037X3, E1037X9, E1039, E104, E1040, E1041, E1042, E1043, E1044, E1049, E105, E1051, E1052, E1059, E106, E1061, E10610, E10618, E1062, E10620, E10621, E10622, E10628, E1063, E10630, E10638, E1064, E10641, E10649, E1065, E1069, E108, E109, E11, E110, E1100, E1101, E111, E1110, E1111, E112, E1121, E1122, E1129, E113, E1131, E11311, E11319, E1132, E11321, E113211, E113212, E113213, E113219, E11329, E113291, E113292, E113293, E113299, E1133, E11331, E113311, E113312, E113313, E113319, E11339, E113391, E113392, E113393, E113399, E1134, E11341, E113411, E113412, E113413, E113419, E11349, E113491, E113492, E113493, E113499, E1135, E11351, E113511, E113512, E113513, E113519, E11352, E113521, E113522, E113523, E113529, E11353, E113531, E113532, E113533, E113539, E11354, E113541, E113542, E113543, E113549, E11355, E113551, E113552, E113553, E113559, E11359, E113591, E113592, E113593, E113599, E1136, E1137, E1137X1, E1137X2, E1137X3, E1137X9, E1139, E114, E1140, E1141, E1142, E1143, E1144, E1149, E115, E1151, E1152, E1159, E116, E1161, E11610, E11618, E1162, E11620, E11621, E11622, E11628, E1163, E11630, E11638, E1164, E11641, E11649, E1165, E1169, E118, E119, E13, E130, E1300, E1301, E131, E1310, E1311, E132, E1321, E1322, E1329, E133, E1331, E13311, E13319, E1332, E13321, E133211, E133212, E133213, E133219, E13329, E133291, E133292, E133293, E133299, E1333, E13331, E133311, E133312, E133313, E133319, E13339, E133391, E133392, E133393, E133399, E1334, E13341, E133411, E133412, E133413, E133419, E13349, E133491, E133492, E133493, E133499, E1335, E13351, E133511, E133512, E133513, E133519, E13352, E133521, E133522, E133523, E133529, E13353, E133531, E133532, E133533, E133539, E13354, E133541, E133542, E133543, E133549, E13355, E133551, E133552, E133553, E133559, E13359, E133591, E133592, E133593, E133599, E1336, E1337, E1337X1, E1337X2, E1337X3, E1337X9, E1339, E134, E1340, E1341, E1342, E1343, E1344, E1349, E135, E1351, E1352, E1359, E136, E1361, E13610, E13618, E1362, E13620, E13621, E13622, E13628, E1363, E13630, E13638, E1364, E13641, E13649, E1365, E1369, E138, E139 |
| Cardiogenic shock | diagnoses_icd during index hospitalization | 78551 | R570 |
| Acute heart failure | diagnoses_icd during index hospitalization | 42821, 42831, 42841 | I5021, I5031, I5041, I50811 |
| Cardiac arrest | diagnoses_icd during index hospitalization | 4275 | I46, I462, I468, I469 |
| Atrial fibrillation | diagnoses_icd during index hospitalization | 42731 | I48, I480, I481, I4811, I4819, I482, I4820, I4821, I489, I4891 |
| Ventricular fibrillation | diagnoses_icd during index hospitalization | 42741 | I490, I4901 |
| Ventricular tachycardia | diagnoses_icd during index hospitalization | 4271 | I472 |
| STEMI/NSTEMI-related flag (st_nst) | diagnoses_icd during index hospitalization | None specified | I210, I2101, I2102, I2109, I211, I2111, I2119, I212, I2121, I2129, I213 |

Supplementary Table S2. Candidate covariates considered for model building

The initial candidate covariates considered for model construction included demographic variables, comorbidities and acute clinical conditions, laboratory parameters, and treatment-related variables. These variables were extracted from the MIMIC-IV database based on clinical relevance and data availability.

| **Demographic variables:** |
| --- |
| Age |
| Sex |
| Race |
| **Comorbidities and acute clinical conditions:** |
| Hypertension |
| Diabetes mellitus |
| Acute heart failure |
| Cardiogenic shock |
| Cardiac arrest |
| Atrial fibrillation |
| Ventricular fibrillation |
| Ventricular tachycardia |
| **Laboratory variables:** |
| Creatinine |
| Glucose |
| Hemoglobin |
| Potassium |
| Lactate |
| Sodium |
| NT-proBNP |
| Platelet count |
| Troponin T |
| White blood cell count |
| **Treatment-related variable:** |
| Percutaneous coronary intervention (PCI) |
| **Additional extracted variable:** |
| Body mass index (BMI) |

Note: BMI was extracted during data processing but was not retained in the final integrated analytic dataset, because of data completeness. Vasopressor use was considered clinically relevant during study design; however, this may reflect the additional complexity of defining time-specific vasopressor exposure in relation to BPV during the first 24 hours after ICU admission, so it was not extracted into the final analytic dataset and therefore was not included in the present analysis.
